# Understanding barriers to men’s support for family planning in rural Ethiopia—findings from the USAID Transform: Primary Health Care Project Gender Analysis

**DOI:** 10.1186/s12978-022-01384-z

**Published:** 2022-06-13

**Authors:** Dustin Andrew Smith, Heran Abebe Tadesse, Kidest Lulu, Diana Santillán

**Affiliations:** 1 EnCompass LLC, 1451 Rockville Pike, Suite 600, Rockville, MD 20852 USA; 2 EnCompass Ethiopia, Nifas Silk Lafto Sub City, Woreda 03, P.O. Box 16982, Addis Ababa, Ethiopia; 3 Pathfinder International Ethiopia, Nifas Silk Lafto Sub City, Woreda 03, P.O. Box 12655, Addis Ababa, Ethiopia

**Keywords:** Ethiopia, Amhara, Tigray, Oromia, SNNPR, Male engagement, Family planning, Antenatal care, Primary health care, Gender equality

## Abstract

**Background:**

Evidence suggests that supportive male engagement in health care services, including family planning, remains low in many countries, despite known benefits for female partners. In 2017–2018, the United States Agency for International Development Transform: Primary Health Care Project conducted a participatory gender analysis, collecting relevant data to better understand Ethiopian men’s lack of support for the uptake of family planning services.

**Methods:**

Qualitative data were collected through 96 unique participatory group discussions with community members via a semistructured discussion guide and participatory activity; data were disaggregated by sex, age, and marital status. In-depth interviews (91) conducted with service providers, health system managers, and health extension workers used semistructured guides. Discussants and interviewees were selected purposefully, drawn from 16 rural *woredas* in four project regions: Amhara; Oromia; Tigray; and Southern Nations, Nationalities, and Peoples’ Region. Data collectors took notes and transcribed audio recordings. The research team deductively and inductively coded transcripts to develop preliminary findings later validated by key technical project staff and stakeholders.

**Results:**

Findings reinforce existing knowledge on the dominant role of men in health care–related decision making in rural Ethiopia, although such decision making is not always unilateral in practice. Barriers at the societal level impede men’s support for family planning; these include norms, values, and beliefs around childbearing; religious beliefs rooted in scriptural narratives; and perceived adverse health impacts of family planning. Lack of efforts to engage men in health care facilities, as well as the perception that health care facilities do not meet men’s needs, highlight systems-level barriers to men’s use of family planning services.

**Conclusions:**

Findings indicate several opportunities for stakeholders to increase men’s support for family planning in rural Ethiopia, including systems-wide approaches to shape decision making, social and behavior change communication efforts, and additional research and assessment of men’s experiences in accessing health care services.

## Plain language summary

Evidence suggests that in instances where men participate when their partners access health care services, their partners experience positive health benefits. Regardless, men tend not to participate. During 2017–2018, the United States Agency for International Development Transform: Primary Health Care Project conducted research to identify gender-related issues that hinder the delivery of primary health care services in Ethiopia. The research team conducted 96 group discussions with male and female community members, as well as 91 in-depth interviews with health care service providers, health system managers, and health extension workers. Participants were specifically selected from 16 rural districts, or *woredas*, in four regions where the project is active. The researchers then categorized information in the resulting transcripts by common themes, and the data analysis team met to draw out the main findings. Later, a meeting was held with key project staff and stakeholders in Addis Ababa to verify the findings. Findings reinforce existing knowledge on the dominant role of men in health care–related decision making for households in rural Ethiopia, although women often play an important role as well. The research also identified widespread male opposition to family planning due to norms, desires, and societal perceptions around childbearing; religious beliefs; and concerns about the perceived health risks of family planning methods. Further, findings showed that the promotion of family planning methods and services do not explicitly target men, and men believe that current services do not respond to their needs. Respondents suggested opportunities for stakeholders to mitigate these barriers.

## Background

An existing body of evidence suggests that supportive male engagement in health care services has a positive impact on myriad health outcomes for their female partners. Several global, systematic reviews of health care–related male engagement interventions indicate improved outcomes in the areas of family planning, maternal nutrition, antenatal care (ANC) attendance, birth and complications preparedness, skilled birth attendance, births taking place in facilities, postpartum care, and reduced postpartum depression [1, 2]. Such improvements are likely indicative of the often disproportionately influential role of men in reproductive, maternal, and child health outcomes [3]. As such, male engagement is critical for addressing gaps in health care service use, including the uptake of family planning methods; research indicates that men better support contraceptive use and shared decision making when they have received relevant counseling [4].

Factors underlying low levels of preexisting male engagement are similar by country and regional context. For instance, research in Nepal suggests that barriers to supportive male engagement include sociocultural and psychological norms, a lack of education, and the predominance of female health care providers in clinics that provide maternal and child health care services [5]. In Kenya, research found that pervasive gender norms and the implementation approaches of reproductive health and family planning programs heavily influenced levels of male engagement [6].

In Ethiopia, men often oppose their partner’s use of family planning methods [7–11]; fail to participate in antenatal care [12–14]; and perceive health care services as unfriendly [15, 16]. Limited research speaks to the factors influencing such behaviors. In a qualitative case study in Southern Ethiopia, the influence of culture, religion, and the perception of family planning as a women’s issue were some factors identified as significant impediments to male involvement in family planning [8]. In Harari State, women cited men’s belief that ANC is primarily their partners’ concern and feelings of shame in accompanying their partners during visits as reasons for their lack of involvement in ANC [17].

Although such findings are informative—and in particular, suggest low levels of male engagement in family planning—the body of evidence on barriers to male engagement in Ethiopia remains nascent. Despite significant progress in recent years, the country experiences persistently high rates of morbidity and mortality due to preventable causes, including maternal and newborn illnesses. Family planning plays a critical role in reducing maternal mortality, as a generally lower number of pregnancies inherently reduces the incidence of medical complications in a given population, and it is an important tool for women to time their births, both in terms of spacing and ensuring that birthing occurs at a medically appropriate age [18]. Further, it reduces the likelihood of a woman accessing an unsafe abortion [18].

Male engagement is a critical component for addressing these gaps and identifying barriers to supportive male engagement is essential for development practitioners, the Government of Ethiopia, and other key stakeholders as they design and implement strategies and interventions to improve health care outcomes. In particular, the Government of Ethiopia has recognized the importance of male engagement in national policy and guidelines, having promoted male engagement in the *National Reproductive Health Strategy* (2006–2015) and the *National Guideline for Family Planning Services in Ethiopia* (2011), the latter stating that men should be thoroughly engaged in family planning programs and services [19].

Drawing upon qualitative data collected during the Transform: Primary Health Care Project Gender Analysis [20], this paper seeks to contribute to the existing knowledge base on barriers to male support for family planning in four regions of Ethiopia: Amhara; Oromia; Tigray; and Southern Nations, Nationalities, and Peoples’ Region (SNNPR). Insights gained will serve to inform approaches for future male engagement efforts in these regions and, ultimately, contribute to reducing preventable maternal and child deaths, moving Ethiopia closer to achieving the United Nations’ Sustainable Development Goals 3 and 5.

The Transform: Primary Health Care Project is a five-year, United States Agency for International Development (USAID)–funded effort that provides technical assistance to the Government of Ethiopia. The project specifically supports the implementation of the government’s *Health Sector Transformation Plan*. Further, the project aims to address reproductive, maternal,newborn, child, and adolescent health and nutrition (RMNCAH-N) in the aforementioned four regions of Ethiopia. The project also recognizes that gender is a key social determinant of health and seeks to integrate gender-transformative activities throughout its implementation.

To date, the Transform: Primary Health Care Project, in coordination with the Ethiopian Federal Ministry of Health, has also undertaken several efforts to bolster men’s acceptance and uptake of modern family planning methods. Recognizing the influential role of health extension workers in health care–related decision making, the Transform: Primary Health Care Project conducts orientation sessions on male engagement approaches for those health extension workers and prioritizes social and behavior change communication efforts in active project regions by producing numerous materials that stress the role of men in RMNCAH-N. The Transform: Primary Health Care Project also conducted capacity-building workshops for religious leaders; the workshops emphasize messaging on RMNCAH-N and their associated gender implications.

To support its gender integration efforts, the project conducted a comprehensive gender analysis in 2017–2018, with the overarching research question *“*What gender gaps and opportunities does the Transform: Primary Health Care Project need to address to achieve its intended results?” This question and corresponding research sub-questions were developed in consultation with project staff and key stakeholders during a design meeting held in Addis Ababa in June 2017. This paper summarizes the gender analysis findings related to men’s engagement in family planning.

## Methods

### Study design

The Transform: Primary Health Care Project gender analysis included a review of relevant secondary data from published sources, and primary qualitative data collection in Amhara, Oromia, Tigray, and SNNPR. The primary objective of the gender analysis was to broadly assess gender equity in the Ethiopian primary health care system—in order to inform a comprehensive strategy for the Transform: Primary Health Care Project. As such, the qualitative data collection tools included questions about the role of men in health care–related decision making and the uptake of family planning, among other key areas.

The qualitative study design was premised on the use of thematic analysis, in order to ensure that the research effectively identified and organized participants’ views and experiences as they help to elucidate a response to the aforementioned research question [21]. In support of this design, appreciative and participatory approaches informed all data collection efforts. All data collection tools were piloted and subsequently revised as part of a comprehensive training held for data collectors in October 2017. Data collection was conducted during November and December 2017 in 16 rural *woredas* of the aforementioned regions, and analysis was conducted in Rockville, Maryland, and Addis Ababa in February and March 2018. Research findings were validated in Addis Ababa in May 2018.

Qualitative data collection included the use of two data collection approaches: *in-depth interviews* and *participatory group discussions*. In-depth interviews—held with health care providers, health facility managers, health extension workers, government representatives from *woreda* and zonal health offices, and representatives from the Women and Children’s Affairs Office—were semistructured and included appreciative questions. Relevant to this study, interview guides for health extension workers included questions pertaining to the reasons that men and boys access health care facilities; how they perceive men to understand and define quality health care; and men’s general engagement in family planning and maternal, newborn, and child health care in their facility. In addition to covering these topics, interview guides for health care providers and health facility managers also contained questions relevant to equity in the provision of health care services for men and women in their facilities.

Participatory group discussions—an innovative approach to conducting traditional focus groups—used semistructured discussion guides with appreciative questions; community mapping; and a unique Paving Stones activity, which drew upon visual aids to help participants identify health resources as well as gender gaps and opportunities in the provision of health care services within their communities. The participatory group discussion guides included a series of 16 questions covering health practices; access to and supports for accessing health care services; and experiences while utilizing such services, including perceptions of quality.

For the purposes of the Paving Stones activity, a group facilitator drew a number of paving stones on a blank flip-chart; based on inputs from participants, each paving stone was inscribed with something that aids or assists them in accessing health care services in their community. Ultimately, these paving stones formed a pathway that would successfully lead them to accessing health care facilities and their available services.

The research team held participatory group discussions with married and unmarried women and men within multiple age groups: (a) married women ages 15–24; (b) married women ages 25–45; (c) unmarried women ages 15–24; (d) married men ages 15–24; (e) married men ages 25–60; and (f) unmarried men ages 15–24. Due to the demographic features of the target regions, where men tend to marry later and remain fecund until a later age, the older groups of married men included a broader age range than the corresponding female groups. The team convened groups of six to eight participants, which allowed for capturing unique health care–related needs and behaviors associated with differences in sex, age, and marital status.

The research team held in-depth interviews and participatory group discussions in each of the four regions the study targeted. Overall, data collection was conducted in two high- and low-performing *woredas* in Amhara, Oromia, and SNNPR, as well as one high-performing and two low-performing *woredas* in Tigray. *Woredas* are deemed high- or low-performing based upon their performance in key RMNCAH-N indicators. For the purposes of this study, *woredas* were specifically selected in consultation with representatives from regional health bureaus and the project’s regional technical coordinators to ensure reasonable representation of the regions’ sociocultural and religious diversity, variations in gender norms, and differences in access to health care services. To support logistical needs, all selected *woredas* were accessible from zonal towns. Within each *woreda*, data were collected within one *kebele* administrative sub-division.

### Study procedures

#### Recruitment

In consultation with local representatives, the Transform: Primary Health Care Project’s regional gender officers purposefully recruited interviewees and group discussion participants in advance of data collection. Participant selection was informed by government or health facility data to the extent possible, and sociocultural variation was taken into consideration when forming discussion groups. Data collectors conducted recruitment in a face-to-face manner, speaking with potential participants using a predefined script. The research team also strived to recruit an equal number of male and female interviewees, to the extent possible.

#### Data collection and sample size

The research team conducted a comprehensive training for data collectors in October 2017. Prior to starting data collection, the team received ethical approval from EnCompass LLC’s internal Institutional Review Board committee and each region’s respective ethical review committee.

Between November and December 2017, the research team conducted 91 in-depth interviews and 96 participatory group discussions (see Tables 1 and 2 below for a disaggregated sample), for a total of 187 data collection events.

**Table 1:** In-depth interviews by region

Region	Providers		Managers		Health extension workers	Government representatives		Total
	Female	Male	Female	Male	Female^1^	Female	Male	
Amhara	4	4	1	3	4	3	5	24
Oromia	5	7	0	6	6	3	4	31
SNNPR	4	4	0	4	4	4	4	24
Tigray	2	2	0	2	2	2	2	12
Total	15	17	1	15	16	12	15	91

^1^Few health extension workers are male; they mostly reside in regions that fall outside the scope of this research effort. As such, all participating health extension workers were female

**Table 2:** Participatory group discussions with community members, by region

Region	Married female		Married male		Unmarried female	Unmarried male	Total
	Ages 15–24	Ages 25–45	Ages 15–24	Ages 25–60	Ages 15–24	Ages 15–24	
Amhara	4	4	4	4	4	4	24
Oromia	6	6	6	6	6	6	36
SNNPR	4	4	4	4	4	4	24
Tigray	2	2	2	2	2	2	12
Total	16	16	16	16	16	16	96

Both in-depth interviews and participatory group discussions were conducted by a combination of regional staff members from EnCompass LLC and external consultants. Teams consisted of one interviewer or facilitator and a note taker. Male data collectors interviewed male key informants and facilitated groups with male participants. Similarly, female data collectors interviewed female key informants and facilitated groups with female participants. Data collectors were selected based on previous academic qualifications in relevant social sciences and a demonstrated interest in issues pertaining to gender equality.

Interviews were held in private rooms or offices in health care facilities, and participatory group discussions were held in mutually agreed upon community locations that were accessible to the participants. No other individuals were present during data collection aside from the approved data collectors and selected participants.

Data collectors provided all research participants with an overview of the data collection process and research objectives as part of the informed consent process. As previously noted, during each data collection event, one data collector was tasked with completing written notes. Interviews and group discussions were both recorded using electronic recorders, to fill gaps in electronic transcription of the notes as needed. At the end of each data collection event, a summary of the conversation was read to all participants, who were asked to confirm the accuracy of the summary and suggest any necessary corrections. In the data collection protocols, data saturation was not prescribed as a criterion for data collectors to continue or end a conversation. Each in-depth interview lasted approximately 90 min on average, and each participatory group discussion lasted approximately 120 min on average.

Preceding data analysis, the research team conducted quality assurance checks of transcripts to ensure the completeness and coherence of content. If gaps or inconsistencies occurred, the respective data collectors were requested to correct them using their audio recordings. The researchers subsequently reviewed any revised transcripts a second time before inclusion in the data analysis process.

#### Data analysis

Qualitative data analysis was conducted between February and March 2018. The research team used Dedoose Version 7.0.23, a web-based data management and analysis application, to both deductively and inductively code approved, translated transcripts. The process was guided by the use of a detailed codebook, which, during the first round of coding, included thematic codes defined in advance of the coding process, based upon pre-identified information that the research team deemed essential for answering the initial research questions. Using a codebook based on themes emanating from the first round of coding, the second round of coding was inductive. Eight coders engaged in this process; consistency amongst coders’ efforts was ensured through pilot tests, in which coders applied the deductive and inductive codebooks to the same transcripts and then convened to identify, review, and respond to any discrepancies in the application of thematic codes. Further, throughout the process, the data analysis manager conducted periodic spot checks to ensure that thematic codes were being applied in accordance with the definitions specified in the codebook.

Subsequently, the team held a participatory data analysis and interpretation meeting in March 2018 to triangulate themes emanating from the data across the various stakeholder groups and generate draft findings. Four members of the research team presented draft findings to project staff and key stakeholders in a data consultation meeting in Addis Ababa on May 16–17, 2018. During the meeting, stakeholders had the opportunity to validate and interpret findings, draw conclusions, and devise recommendations to support the Transform: Primary Health Care Project’s gender integration efforts. Individual team members were tasked with collating feedback from each session held in the meeting; one team member was tasked with integrating feedback into the working version of the findings, and other technical staff provided inputs.

## Results

A total of 187 data collection events with interviewees and group discussants satisfied the purposeful sample previously described. Qualitative data from the 91 in-depth interviews with health care providers, health facility managers, and health extension workers, as well as from the 96 participatory group discussions held with unmarried and married men and women from both age groups provide insights on a number of barriers to male engagement in family planning. Many of these barriers are rooted in societal views, norms, and beliefs, whereas others represent systems-level limitations within the Ethiopian primary health care system.

Below, using these categories, we discuss the gender analysis findings on barriers to male support for family planning. Unless indicated otherwise, references to findings from respondents, key informants, and focus group discussions are the result of the triangulation of key themes across the various stakeholder groups, which occurred during data analysis.

### Societal views, norms, and beliefs

Within the realm of societal views, norms, and beliefs, the influence of norms, desires, and societal perceptions around childbearing are particularly influential, as are religious beliefs and the perceived health risks of family planning methods. Such barriers are heavily intertwined and embedded in the partner dynamics of individuals who reside in the rural regions targeted for the initial Transform: Primary Health Care Project Gender Analysis.

#### Partner dynamics of health care decision making in rural Ethiopia

Participatory group discussion participants across all four regions and age groups (except male participants in both age groups from Tigray) acknowledged that men were the primary, and sometimes sole, decision makers with regard to health care–related matters for themselves and their families. In all regions, female group discussion participants, ages 15–24 and 25–40, indicated that men—primarily husbands and fathers—influenced their decisions on when and where to access health care services in general.

More specifically, female participants in both age groups in Amhara, Oromia, and Tigray, as well as women ages 25–40 in SNNPR, said men influenced their health care–related decisions in discussions or by providing advice. Female participants, ages 15–24 and 25–40 in Amhara and Oromia and ages 15–24 in SNNPR and Tigray, also spoke about men having full authority to make the “ultimate decision” on matters related to their health care. For instance, one female participant reiterated this finding:

As a tradition, the head of the household, which is most [of] the time men, make[s] decision and the rest of the family members adhere to that decision. If our fathers say let’s try traditional medicine, our mothers will try it. The opposite is also true, if our fathers say let’s go to the health center, then we will go to the health center. Such tradition is somehow altering, in some families, father and mother discuss and decide together, but in some families it’s the father that have [sic] the final say when it comes to deciding over whether one family member should go to the health center or not. —Female participant, group discussion with unmarried women ages 15–24, Tigray

Respondents across regions also frequently stated that men supplied other support critical for their access to health care services, such as financial support. In Amhara (women ages 15–24), Oromia (women ages 25–40), and Tigray (women ages 14–25 and 25–40), group discussion participants talked about the importance of men assisting them with transportation or chaperoning them to health facilities. Several female participants expressed the financial role that men play in their access to health care services:

As it has been said, majority of women don’t have income of their own, hence our husbands are the ones who pay money for community-based health insurance and also for our medical bill. Hence, they help us to make decision by giving us the money and also by going with us if our disease is severe. —Female participant, group discussion with married women ages 25–40, Tigray

Even when I want to go to a health facility today, he says that he can’t take me today. And I get mad. I feel that that is happening because I am dependent on them and because I don’t have money. —Female participant, group discussion with unmarried women ages 15–24, Amhara

Although such findings indicate that men have the ultimate authority in making health care–related decisions, data suggest this is not an entirely unilateral process. Across age groups, male and female discussion participants described a process for health care decision making that included spouses, parents, neighbors, or other community members and that took into account advice from health extension workers or other community groups. Deeper conversation during the participatory group discussions also revealed that unmarried and married females from Amhara, SNNPR, and Tigray felt supported in their decision making, although many expressed frustrations with their often-limited degree of autonomy. Further, in Amhara and SNNPR, more young unmarried male respondents ages 15–24 than female group discussion participants expressed their displeasure and unhappiness with the involvement of other individuals in their health care–related decision making.

In health care decision making around the use of family planning methods, married men and women ages 25–40 explicitly indicated the general prevalence of men’s opposition to the use of family planning, which often led women to seek such services in secret. In particular, female discussion participants in both age groups (15–24 and 25–45 years) in Amhara and Oromia frequently noted that their husbands’ opposition to family planning required them to access services without their husband’s knowledge. Men’s reasoning for such opposition varied by region but included norms, desires, and societal perceptions around childbearing; religious beliefs; and perceived adverse health effects resulting from the use of family planning methods. These are subsequently discussed in turn, below.

#### Influence of norms, desires, and societal perceptions around childbearing

Men in both age groups in Amhara and Oromia often expressed a strong desire for additional children, regardless of whether such desire aligned with the views of their partners; this desire precludes the use of family planning methods. This matter resonated among many female participants:

Nevertheless, since quite majority of our rural men [are] proud of having as many children as they could, while women have least interest of having more. In this case, most women fearing their husband’s outright objection of using family planning, they went to the health facilities by their own. They also keep its confidentiality. —Female participant, group discussion with married women ages 25–40, Oromia

In the locality, men have a bias of considering children as an asset and wanted to have more children. Old women and some married women of my age are not allowed to take family planning services to control births. Thus, when they want to receive family planning services secretly, they prefer to go either alone or along with friends. —Female participant, group discussion with married women ages 25–40, Oromia

Some participants, including married women ages 25–40 in Oromia, a health facility manager in SNNPR, and a health service provider in Amhara, noted that underlying this desire is the fact that children are often considered an asset to their families. Some female discussants from both age groups in Oromia indicated that it is also specifically reinforced by in-laws, who hold certain expectations about childbearing. Two female participants noted the following:

My fellow friends and relatives informed me that my husband’s family likes to see me giving birth to as many children as possible and do not want me using family planning services. —Female participant, group discussion with married women ages 15–24, Oromia

My husband and his families wanted me giving more births and objected the use of birth control methods. —Female participant, group discussion with married women ages 15–24, Oromia

In Amhara and Tigray, some key informants indicated that among the most significant reasons behind men’s desire for more children was an ingrained fear that their spouse could leave them if they were unable to father additional children. As a result, some men decline to use family planning services. An interviewed health extension worker noted the following:

There are some people who are not well aware and still think that their wives will leave them if they don’t have many children. —Health extension worker, Tigray

Despite such opposition to the use of family planning, whether rooted in social norms or other accepted societal beliefs, some key informants from Amhara, Tigray, and SNNPR suggested that there was a small but increasing desire among men to limit their family size. Some of those men actively supported their wives in the use of family planning methods. Respondents noted that this shift was more prevalent among younger age groups and driven by economic realities, including the general cost of living and limited resources to sustain family life. Numerous key informants identified this shift, including a government representative from Amhara and a health extension worker from Tigray:

Previously, they do think that their wives need to keep giving birth as long as they are their wives. Now, the life itself is influencing them so that now they are communicating how many children and when to have them. —Government representative, Amhara

Young men have grasped the idea that having many children is not an asset. It will endanger the mother’s health as well as the children’s. An unplanned child also creates economic crises in the household; thus, the child will not get balanced diet, and this will in turn affect the intellectual capabilities of the children. —Health extension worker, Tigray

Similarly, some female discussion participants reported that their husbands were open to and encouraged family planning. Interviewees in Amhara, Oromia, and SNNPR echoed this by citing family planning as one of the main reasons that men access health care. Interviewees also noted men’s willingness to visit health facilities with their partners to use family planning services. One young female participant mentioned the following:

Sometimes, husbands prohibit their wives to take contraceptives. At that time, the women take the contraceptive method in secret. But also, there are some who permit to do so. I, for example, took family planning by consulting my spouse. He suggested to me to take contraceptive method because he wants me to continue my grade 10 education. We have a five-year-old baby son. —Female participant, group discussion with married women ages 15–24, Amhara

#### Influence of religious belief on family planning

In all regions, religious belief was frequently cited as a major reason for men’s opposition to the use of family planning; among participatory group participants, this response was most pronounced among married male respondents ages 25–40. Both Christian and Muslim participants cited scripture regarding the belief that God created the earth and commanded human beings to procreate and fill it in abundance. Therefore, family planning was seen as disrespectful to God, and some considered disrupting procreation to constitute sin. During in-depth interviews, participants described a number of instances where husbands who identified themselves as religious adherents had asked service providers to remove Implanon, an implanted birth control method. A health extension worker expressed his awareness of the influence of religion in men’s acceptance of family planning methods:

Some religious leaders are perpetuating this way of thinking; they say using

contraceptive is a sin and a woman need to have as many children as possible until

God makes her infertile. —Health extension worker, Tigray

Further, one informant indicated that religious beliefs were so influential in her community that some men who actively supported their partner’s use of family planning methods were compelled to pretend, in front of other community members, that their wives did not use such methods:

They say that it is not allowed by *Sheria* [Muslim religion doctrine]. They don’t want it that much and they don’t want to be talked about it. Even if he wants to use, even using in secrecy, he doesn’t make it open to others/doesn’t tell to others. —Health extension worker, Amhara

#### Perceived health risks of family planning methods

Study participants also noted the perceived health risks of family planning methods were a key obstacle to men’s uptake of family planning. Across all four regions, married men ages 25–40 expressed their concerns with the potential health risks of family planning methods, especially injectables. Men cited a fear that these methods could lead to long-term infertility for their partner and often did not advise their wives to use family planning. Among those who supported their spouses in the use of family planning, some expressed suspicions or uncertainty about the side effects of certain methods. Several key informants expressed their awareness of these perceived risks within the communities they serve:

The men reason out that the wives could fall sick if they practice contraceptive methods like that of injectable. …Some women who practice family planning methods without knowledge of their husbands will take care to place the patient card in a location that the husbands could not reach at. —Health service provider, SNNPR

The husbands’ motive is clearly to get children because they assume that contraceptive use over long time would cause complication to child delivery or sterility. —Health facility manager, Amhara

Wives practice family planning at all [sic]. They say that they like that, their wives give birth for more children. The men reason out that the wives could fall sick if they practice contraceptive methods like that of injectables. —Health service provider, SNNPR

### Systems-level limitations within the Ethiopian primary health care system

Several of the identified barriers reflect the presence of systems-level limitations within the Ethiopian primary health care system—namely, the inequitable targeting for health services and family planning promotion. The identification of such barriers reinforces the need for bolstering male engagement efforts within the Ethiopian primary health care system and suggests possible entry points for future action.

#### Inequitable targeting for health services and family planning promotion

Data indicate a number of health systems–level concerns regarding the promotion and delivery of family planning services. Namely, there is a perception that men are explicitly not targeted in the promotion of family planning methods and services; further, respondents expressed the belief that current health services do not respond to men’s needs. However, it is also worth noting that for group discussion participants who did access family planning services, including counseling and the options available, the majority expressed satisfaction. In particular, participants expressed their satisfaction with family planning services provided by health extension workers.

Some key informants from Amhara pointed out there was gross ignorance among men with regard to family planning. They also noted that health education sessions on family planning were presented every day for those who visited the health facilities, yet such education was not tailored to men. The fact that women used the majority of family planning methods was cited as one factor driving the exclusion of men from such programming. They articulated that while decision making around family planning use was mainly influenced by men, promotion strategies continued to target women. They also noted numerous missed opportunities to engage men to promote family planning. For instance, they mentioned that while health facilities regularly conducted conferences for pregnant women^1^ and discussion groups for mothers, these efforts did not seek to include men.

I think this is related to the misconception that reproductive health and maternal health care issues are regarded as women’s problems. —Health facility manager, Amhara

Across all regions, approximately half of both male and female discussants perceived that services were provided in an inequitable manner in health facilities, based on a number of factors. These factors included dress, educational qualifications, and socioeconomic status, among others. The male discussion participants also specifically cited discrimination in treatment due to patients’ personal connections with providers; for instance, they believed that the family and friends of providers were granted priority in receiving care.

Some young unmarried men, across all regions except Tigray, also felt dissatisfied with existing health care services; they named a lack of education on the use of condoms, family planning services, and HIV testing services as contributing to their dissatisfaction. Overall, they described that the provision of these critical health care services was not tailored to their needs as youth. In particular, health care providers in Amhara, Oromia, and Tigray, as well as participants in one group discussion with young unmarried males in Oromia, discussed youth friendly services; mentions of youth friendly services occurred without prompting by interviewers or facilitators. Such providers noted that with regard to a number of sensitive topics, including sexual histories and reproductive health care needs (e.g., condoms and instruction on their use, HIV testing), youth appeared reluctant to have such conversations. One young male participant in SNNPR expressed his frustrations as the following:

In my evaluation, I was afraid to say their service is good, since they have no any [sic] means to educate youths like me, there is no condom in a free space, and they are not teaching the people about HIV/AIDS. For instance, last week, I and my friends came to the health center to get condoms, but it is only the carton—you can see no condoms inside, so how is it possible to say it is good? —Male participant, group discussion with unmarried men ages 15–24, SNNPR

#### Identified need for bolstering male engagement and possible entry points

Furthermore, corresponding with the general finding of male opposition to family planning, several respondents in various groups—including both female group discussion participants; health care staff (managers, providers); and health extension workers—articulated that additional efforts were needed to bolster male involvement in not only family planning but also health care–related conversations and education more broadly. They made recommendations for improving men’s knowledge of modern family planning methods and their associated benefits, with the understanding that increased knowledge would improve male participation in this realm. Female discussion participants and key informants referenced the broad need for increased male engagement in family planning at the community level. Among possible entry points, two health facility managers suggested engaging men on the topic of family planning during antenatal appointments and while men access outpatient services. Health extension workers, as well as *kebele* officials, were also noted as potentially playing a significant role in engaging men in family planning.

## Discussion

Findings from this research described partner dynamics in rural Ethiopia and highlighted a number of barriers to male engagement in family planning. Some barriers are rooted in societal views, norms, and beliefs, including the influence of norms, desires, and societal perceptions around childbearing, religious beliefs, and the perceived health risks of using family planning methods. Findings at the health systems level identified that health services and family planning promotion inequitably target users and that such services are perceived as being unfriendly to men. These findings corroborate and contribute to a nascent body of literature from Ethiopia on barriers to male support for family planning.

The initial exploration of partner dynamics substantiates preexisting ideas around men’s disproportionately influential role in reproductive, maternal, and child health [3]. In the context of four regions of Ethiopia, data indicate that men provide numerous forms of support that are often critical for women’s access to health care services, including financial support, transportation, and chaperoning those traveling for care. Husbands and fathers are often considered the primary and, sometimes, sole decision makers about their own and their family’s health care.

In line with several other studies in Ethiopia [7, 9], data suggest that decision making is not an entirely unilateral process among partners. As part of this study, participatory group discussion participants—both male and female—described a process for health care decision making that included numerous other stakeholders, including parents, neighbors, community members and groups, and health extension workers. This finding suggests the potential benefit of systems-wide approaches that involve other influential stakeholders to positively shape health care–related decision making in rural Ethiopian households.

Findings from this study on the influence of norms, desires, and societal perceptions around childbearing are also corroborated by existing literature from Ethiopia. Numerous studies cite a desire for more children as a key driver for men’s opposition to the use of family planning services [7–11]. In particular, one study also captured men’s concerns around the stability of their marriage or partnership with regard to childbearing; some men believed that childbearing reduces their partner’s potential for committing acts of infidelity [8].

This research also expands upon the existing knowledge base on the important roles of both religious beliefs about and the perceived health risks of family planning methods in dissuading men from utilizing family planning methods. Adherents of both Christianity and Islam, the predominant religious groups in the regions studied, stated religious belief forbade the use of family planning; this finding contributes to the existing knowledge base on the influence of religion on men’s attitudes toward family planning [8, 9, 11]. Respondents from this study, including married men in all regions, also expressed widely held convictions that modern family planning methods posed adverse health risks for women, including long-term infertility; these fears often centered on injectable methods such as Depo-Provera. Such fears have been previously cited in other studies in Ethiopia [7, 9, 11].

The persistence of these barriers suggests a number of opportunities for social and behavior change communication efforts to shape norms around childbearing and family size, as well as more general educational outreach activities to make sure men understand the innumerable benefits of family planning methods and have accurate information on their associated risks. Considering the dynamic role of religious belief in shaping men’s acceptance of family planning, religious leaders are a highly influential group that could also be engaged to encourage family planning uptake.

Data from this study also reinforce the fact that in many areas of Ethiopia, health services and family planning promotion are perceived to inequitably target users, often concentrating efforts and resources on female clients. Some key informants suggested a general misalignment between efforts to promote family planning and services and their inclusion of men. This gap was not only identified by key informants actively engaged in the operation of health care facilities but also by service users, who in participatory groups suggested that even if men visited facilities with their partners, their presence was likely to go unnoticed. Several other studies in Ethiopia have also stressed the need for better targeting men in the provision of family planning services [7, 9, 10].

Further, although this study found that some male service users feel that health care services are dissatisfactory and often unfriendly to men, this barrier to men’s uptake of family planning services remains largely unexplored in the Ethiopian literature. Additional research, focusing primarily on men’s experiences accessing reproductive health services, as well as other health care services more broadly, would provide deeper insight on this important topic. Such insights could potentially go a long way to assist health care providers and other stakeholders in mitigating this barrier. Further, the perceived misalignment between men’s needs and the provision of services necessitates careful assessments in health care facilities and the communities they serve to secure successful alignment.

It is also worth noting that several limitations influenced findings from this qualitative study. The data collection sample did not engage older unmarried women and men, older than age 24, who are a minority because of the demographic features of the target regions where early and child marriage is prevalent. Further, although the research team sought to recruit an equal number of male and female key informants, recruiting an equitable number of male and female health facility managers was challenging because of women’s generally low representation in such roles throughout Ethiopia.

Additionally, the categorization of some *woredas* as high- or low-performing shifted during data collection, and those shifts were not captured during the data analysis phase. In addition, due to civil unrest, a different *woreda* was sampled in Oromia region than originally planned. The team also used audio recordings to supplement written transcripts; in instances where recordings failed, omissions in transcripts had to be recovered via data collectors’ individual recollections. Furthermore, despite efforts to minimize bias in recruitment, the participation of local “gatekeepers” in selecting informants and group participants might have skewed the resulting set of participants.

In spite of such limitations, the depth of findings captured in this study serves to suggest the utility of the unique approach to data collection that was used. Although the use and triangulation of data captured through in-depth interviews and group discussions are commonplace in contemporary qualitative research, the introduction and use of participatory group discussions, integrating the aforementioned Paving Stones activity, likely enabled the participants to effectively shape and lead the conversation in ways that helped the researchers to identify the unique barriers to men’s support for the use of family planning in their communities.

Ultimately, findings from this research effort undoubtedly serve to corroborate and expand upon the existing but rather limited knowledge base on barriers to male engagement in family planning in Ethiopia. In addition to assessing key partner dynamics, qualitative data revealed nuanced details around the role of societal views, norms, and beliefs in influencing men’s use of family planning services. These center on norms, desires, and societal perceptions around childbearing, religious beliefs, and the perceived health risks of using family planning methods. Findings also serve to clarify the need for additional focus on men in the promotion of family planning services and the general provision of relevant health care services.

## Conclusion

Findings from the Transform: Primary Health Care Project Gender Analysis both support and expand upon existing knowledge on the dominant role of men in health care–related decision making in rural Ethiopia and reinforce the fact that such decision making is not always unilateral in practice; numerous other stakeholders also inform women’s health care–related decision making.

The qualitative data also revealed that, at the societal level, norms, values, and beliefs around childbearing; religious beliefs rooted in scriptural narratives; and perceived adverse health impacts of family planning use impede men’s engagement in family planning. Such views appear largely pronounced across the regions included in the study. Health care facilities’ lack of male engagement in family planning and the perception that health care facilities do not meet men’s needs highlight systems-level concerns that likely hinder men’s use of family planning services.

Overall, these findings suggest a number of opportunities for the Transform: Primary Health Care Project and other concerned actors in Ethiopia to mitigate the identified barriers to supportive male engagement in family planning, including the use of systems-wide approaches to involve other influential stakeholders in positively shaping health care–related decision making in rural Ethiopian households; conducting social and behavior change communications to shape norms around childbearing and family size, as well as imbue upon men an understanding of the risks and benefits of family planning methods; additional research on men’s experiences while accessing reproductive health services; and careful assessments of health care facilities and the communities they serve to ensure alignment between men’s needs and the services they provide.

## Data Availability

Data will be shared upon reasonable request.

## Abbreviations

ANC: Antenatal care
SNNPR: Southern Nations, Nationalities, and Peoples’ Region
RMNCAH-N: Reproductive, maternal, newborn, child, and adolescent health and nutrition
USAID: United States Agency for International Development

## Footnotes

1 Pregnant mothers’ conferences are half-day meetings held at the *kebele* level with pregnant mothers. They provide a platform for healthcare staff to deliver information about ANC, testimonies about the benefits of facility delivery, and ways to identify signs of danger during pregnancy, among other key messages.
